# Basic life support knowledge among healthcare providers in Afghanistan: a cross-sectional study of current competencies and areas for improvement

**DOI:** 10.1097/MS9.0000000000000273

**Published:** 2023-03-16

**Authors:** Arash Nemat, Mohammad Hamid Nedaie, Mohammad Yasir Essar, Henry Ashworth, Hasibullah Aminpoor, Abdul Wahed Sediqi, Wafaa Binti Mowlabaccus, Shoaib Ahmad

**Affiliations:** a Department of Cardiology, Ariana Medical Complex; b Department of Microbiology, Kabul University of Medical Sciences; c Kabul University of Medical Sciences, Kabul, Afghanistan; d Department of Global Public Health, Karolinska institutet Stockholm, Sweden; e Department of Emergency Medicine, Highland Hospital, Alameda Health System, Oakland, California, USA; f Department of Medicine, Northampton General Hospital, UK; g Department of Medicine, District Headquarters Teaching Hospital, Faisalabad, Pakistan

**Keywords:** basic life support, knowledge, demographics, practice, Afghanistan

## Abstract

Basic life support (BLS) is a type of emergency care provided by healthcare workers and public safety professionals to individuals experiencing cardiac arrest, respiratory distress, or other cardiopulmonary emergencies. Despite having a high burden of cardiovascular disease and trauma from conflict in Afghanistan, little is known about the level of BLS knowledge Afghani healthcare workers have. A cross-sectional study was conducted in Kabul, Afghanistan, to assess healthcare workers’ training and knowledge of BLS. The study, which took place from March to June 2022 across multiple public and private hospitals, was approved by the institutional ethics committee of Ariana Medical Complex. The sample size was calculated using a nonprobability convenience sampling method, and the study population consisted of healthcare workers actively working in a health center who were willing to complete a questionnaire. The results of the study showed that most participants (71.3%) were in the 21–30-year-old age range, and a third (32.3%) were doctors. 95.3% of participants had poor knowledge of BLS, with a mean score of 4.47±1.58 out of 13. Additionally, it was evident from questionnaire responses that providers are not adequately performing BLS. These findings suggest that further work, including regular BLS courses, is necessary to improve the knowledge and practice of BLS by healthcare workers in Afghanistan.

Highlights

- Basic life support is a term used to describe the type of treatment that healthcare workers and public safety professionals provide to people who are experiencing cardiac arrest, respiratory distress, or any cardiopulmonary distress during a medical emergency.
- The study population consisted of healthcare workers actively working in a health center regardless of age, sex, or clinical specialty, working as regular employees in their departments and willing to respond to the questionnaire.
- Most participants belonged to the 21–30-year-old category (71.3%), followed by 31–40 (17.2%).
- 95.3% of participants had poor knowledge of basic life support with a mean score of 4.47±1.58 out of 13.

## Introduction

Cardiovascular disease (CVD) is a leading cause of morbidity and mortality in lower-middle-income countries, accounting for 57% of deaths in these countries^1^. In Afghanistan, CVD is the leading cause of death, accounting for 40 195 (17%) of all deaths in 2020. According to the WHO, this led to Afghanistan having the sixth highest rate of CVD deaths in the world in 2020^2^. Additionally, Afghanistan suffers from a double burden of disease due to the ongoing conflict the country faces. Conflict and terrorism have been the leading causes of disability-adjusted life years in Afghanistan over the last decade^3^. Collectively, the high burden of conflict and CVD necessitate the utilization of quality basic life support (BLS) to improve patient outcomes in Afghanistan.

BLS is a term used to describe the emergency care provided by healthcare workers and public safety professionals to individuals experiencing cardiac arrest, respiratory distress, or other cardiopulmonary emergencies^4^. BLS contains a number of interventions, including cardiopulmonary resuscitation (CPR). According to the American Heart Association (AHA), CPR is an emergency lifesaving procedure that attempts to restore circulation and breathing in an individual who has experienced cardiac arrest by providing chest compressions to help circulate blood and oxygen to the body, as well as rescue breaths to help oxygenate the body^5^. It is an essential component of BLS and can be critical in the early stages of an emergency CVD. BLS dictates that in the event of a cardiac arrest, rescuers should call for help, perform CPR to restore coronary and cerebral blood flow, and use an automated external defibrillator to treat ventricular fibrillation or ventricular tachycardia if present. When performed correctly, BLS has been shown to have significantly improved patient outcomes after cardiac arrest, including neurological function and overall mortality^6^.

Therefore, it is essential for healthcare workers and public health professionals to have proper knowledge and practice of BLS and CPR. Delay in initiating essential BLS and CPR procedures can lead to severe neurological complications, death, and increased health expenditure^6^. However, there is currently no information on the extent of BLS training or knowledge of performing BLS in Afghanistan. To address this gap, this cross-sectional study aims to evaluate BLS knowledge, including definitions, indications, and correct performance of CPR, among healthcare workers in Afghanistan. The results of this study will provide valuable insights for health policymakers and may serve as a catalyst for further investigation into ways to improve BLS knowledge and practice in Afghanistan, thus saving more lives in a country disproportionately affected by CVD and conflict.

## Participants and methods

### Study context

A cross-sectional study using the convenience sampling (nonprobability) method was conducted from March to June 2022 among healthcare workers in the public and private hospitals of Kabul, Afghanistan. This study was conducted following the STROCSS guidelines for cross-sectional studies^7^. The participants were included from a number of public and private of Kabul hospitals: Jamhoriat, Ibni Sina, Maiwand, Estiqlal, Barchi, Ali Abad, Aryana, Amiri, Royal, Global, Shaikh Zayid, Wazir Akbar Khan, Indragandi, Bed Khair Khana, Sama, Khatumul Nabeyeen, Milat, Malalai, and Rabeya Balkhi. The study proposal was approved by the institutional ethics committee of Ariana Medical Complex (AMC-RC-31-20211208).

### Study instrument

This study utilized in-person interviews recorded on a paper questionnaire. The first section of the questionnaire consists of demographic and professional details of the participants, including BLS certification, if any. The second part of the questionnaire contained the multiple-choice knowledge related questions adapted from the AHA 2019 BLS Guidelines. The items in the questionnaire were taken from previous studies’/questionnaires found in the literature^8^,^9^. Further validation prior to this survey was performed by adaptation of the survey items with the AHA BLS guideline 2020 and by applying a pilot study (*n*=50). The necessary corrections were made accordingly after consultation with an emergency care specialist. Each question in the survey carried the same weight.

### Sample size and data collection

The sample size was calculated using Raosoft’s sample size calculator with a 5% margin of error and 95% confidence level, which assumed a population size of 300. Using a convenience sampling (nonprobability) method, participants were recruited in person from the hospitals previously listed. Participants were informed of the study protocol and informed consent was taken from the participants prior to participating in the study. Questions were asked in person and data were recorded on paper forms. The study population consist of healthcare workers actively working in a health center regardless of age, sex, and clinical specialty; they were working as regular employees in their departments and willing to respond to the questionnaire. Those working as academicians, undergraduates, and observers were excluded. Data was collected after receiving written consent forms from all the participants.

### Data analysis

Results recorded on the paper responses were manually entered in Excel and then entered and analyzed through IBM Statistical Package for the Social Sciences (SPSS) version 25. A descriptive analysis was conducted using frequencies and percentages. Total scores for knowledge and practice were categorized into two categories: less than 75% as “POOR”, and more than or equal to 75% as “GOOD”. This cutoff point was used as it is closely linked to a passing grade, but is lower than the 80% required by the AHA to pass BLS certification^10^. Association of the items were analyzed using the *χ* ^2^-test.

## Results

From the demographics, most participants belonged to the 21–30-year-old category (71.3%), followed by 31–40 (17.2%), while the rest accounted for 11.6%. As for occupations, roughly a third of them were doctors (32.3%). Slightly over 50% of the participants had less than or equal to 5 years of experience since graduation (54.4%). Half of the participants had seen BLS training done before (50.9%) (Table 1).

**Table 1:** Demographics of Participants

Characteristics	Frequency	Percentage
Age		
21–30	241	71.3
31–40	58	17.2
41–50	25	7.4
51–60	12	3.6
61–70	2	0.6
Occupation		
Doctor	109	32.3
Dentist	113	33.4
Nurse	116	34.3
Years since graduation		
≤5	184	54.4
>5	153	45.6
Have you ever seen BLS training before		
Yes	173	50.9
No	166	49.1

BLS, basic life support.

Above 50% of participants knew that the abbreviation for CPR means cardiopulmonary resuscitation (86.1%). However, 95.3% of participants had poor knowledge with regards to BLS with a mean score of 4.47 out of 13. For instance, only 10.4% of participants knew the depth of chest compression for children while doing CPR and only 4.7% knew the location of chest compression in infants (Table 2).

**Table 2:** Questions Related to Knowledge on BLS

Questions	Correct [*n* (%)]	Incorrect [*n* (%)]
What does the abbreviation mean? “BLS”	70 (20.7)	268 (79.3)
When you find someone unresponsive in the middle of the road, what will be your first response?	200 (59.2)	138 (40.8)
If you confirm somebody is not responding to you even after shaking and shouting at him, what will be your immediate action?	29 (8.6)	309 (91.4)
What does the abbreviation of CPR means?	291 (86.1)	46 (13.6)
What is the location for chest compression?	26 (8.1)	312 (92.3)
What is the location for chest compression in infants?	16 (4.7)	322 (95.3)
How do you give rescue breathing in infants?	143 (42.3)	195 (57.7)
If you do not want to give mouth to mouth, CPR can be done	100 (29.6)	238 (70.4)
What is the depth of chest compression in adults	87 (25.3)	251 (74.3)
Depth of compression in children during CPR	35 (10.4)	303 (89.6)
Depth of compression in neonates during CPR	82 (24.2)	256 (75.7)
What does abbreviation AED stand for?	132 (39.1)	205 (60.7)
What does abbreviation EMS stand for?	22 (6.5)	316 (93.5)
Total knowledge score	4.47/13	
Good knowledge	16 (4.7)	
Poor knowledge	322 (95.3)	

AED, automated external defibrillator; BLS, basic life support; CPR, cardiopulmonary resuscitation; EMS, Emergency Medical Services.

As for practice, with a mean score of 2.29 out of 5, 93.4% of participants were noted to have poor practice knowledge. The majority of participants gave a correct response to only one question regarding the best action forward with an unresponsive adult that was submerged in water (69.2%) (Table 3).

**Table 3:** Questions Related to Practice on BLS

Questions	Correct [*n* (%)]	Incorrect [*n* (%)]
If you and your friend are having food in a canteen and suddenly your friend start expressing symptoms of choking but responsive, what would be your first response?	66 (19.5)	272 (80.5)
You are witnessing an infant who suddenly started chocking while playing with a toy, you have confirmed that he has been unable to cry or cough, what will be your first response?	98 (29.0)	240 (71.0)
You are witnessing an adult unresponsive victim who has been submerged in fresh water and removed from it. He has spontaneous breathing but is unresponsive. What is the first step?	234 (69.2)	103 (30.5)
You noticed that your colleague has suddenly developed slurring of speech and weakness of the right upper limb. Which one of the following can be done?	108 (32.0)	230 (68.0)
A 50-year-old gentleman with retrosternal chest discomfort, profuse sweating, and vomiting. What is next?	69 (20.4)	269 (79.6)
Mean practice score, SD	2.29/5	
Good practice	22 (6.6)	
Poor practice	316 (93.4)	

BLS, basic life support.

Thirty two percent of participants that were doctors generally had good knowledge on BLS compared to dentists and nurses (6.7 and 3.2%, respectively) (*P*=0.002). Additionally, 22.1% of participants who had seen a BLS training had good knowledge of BLS compared to 9.7% of participants who had not seen one before (*P*=0.018). No significant relationship was recorded between good knowledge and age and years since graduation (Table 4).

**Table 4:** Relationship Between Good Knowledge and Demographics

Characteristics	Percentage of Good Knowledge	*P*
Age		
21–30	10.4	0.909
31–40	9.6	
41–50	3.8	
51–60	7.1	
61–70	0	
Occupation		
Doctor	32.0	0.002
Dentist	6.7	
Nurse	3.2	
Years since graduation		
<5	11.3	0.421
>5	10.1	
Have you ever seen BLS training before		
Yes	22.1	0.018
No	9.7	

BLS, basic life support.

30.6% of participants that were doctors generally had good practice on the BLS compared to dentists and nurses (11.9 and 7.3%, respectively) (*P*=0.002). There was no significant relationship between good practice age, years since graduation and previously seeing a BLS training (Table 5).

**Table 5:** Relationship Between Good Practice and Demographics

Characteristics	Percentage of Good Practice	*P*
Age		
21–30	10.0	0.929
31–40	11.5	
41–50	4.2	
51–60	9.1	
61–70	0	
Occupation		
Doctor	30.6	0.015
Dentist	11.9	
Nurse	7.3	
Years since graduation		
≤5	10.8	0.571
>5	8.5	
Have you ever seen BLS training before		
Yes	14.2	0.231
No	10.0	

BLS, basic life support.

## Discussion

The present study is the first cross-sectional study conducted on the knowledge and practice of BLS among healthcare workers (doctors, dentists, and nurses) in Afghanistan. The findings from this study show that Afghan healthcare workers lack critical knowledge for conducting BLS. Both international and national health policymakers must pay critical attention to these findings and conduct training programs for healthcare workers, students, and the general adult population. In a country like Afghanistan, BLS training is essential due to the unstable and insecure sociopolitical situation, conflict, and high rate of CVD. In such a fragile country, it is essential that healthcare providers know how to perform BLS in order to help patients survive.

In the present study, a majority of the participants were between the ages of 21 and 34 and were equally split between nurses (34.3%), dentists (33.4%), and doctors (32.3%). Of all the participants, 50.9% had previously had BLS training before, while 49.1% had not. In a similar study conducted in Nepal among medical professionals at Kist Medical Hospital, 69% of the participants reported no previous BLS training at all after their graduation^11^. Only 22% of the respondents had received some training within the last 5 years. However, in another study conducted in Germany, the majority of the participants stated they had taken part in BLS training^9^.

Regarding the knowledge of BLS, 89.6% of the participants indicated poor knowledge. This is similar to a study conducted in India among young doctors, where a majority of the participants indicated a poor knowledge of BLS^12^. However, after taking a course on BLS, the participant’s knowledge about BLS increased. Training programs on BLS in Afghanistan can also be conducted to improve participant’s knowledge. Furthermore, courses on BLS should be added to medical curriculum to give medical students an exposure of BLS at the early stage of their career. Similarly, in another study conducted in Uganda among undergraduate medical students, only 29.3% of had good knowledge. Prior BLS training and level academic progress were associated with good BLS knowledge^13^.

Different studies have been conducted around the world to determine the general level of knowledge, attitude, and awareness of the BLS in community settings. For instance, the BLS training rate was around 70% in Slovenia, which is considered a high percentage and reflects a particular policy where training is a mandatory part of driving^14^. Similarly, BLS education rates among community members vary worldwide, such as in Washington, DC (78%), Poland (75%), Western Australia (64.1%), Japan (58%), Turkey (40.3%), Ireland (28%), New Zealand (27%), and Hong Kong (21%)^14^. These differences across countries are likely due to economic resources. The differences observed could be due to the demographics and availability of the training and courses in the curriculum. For example, in Afghanistan, where poverty and illiteracy are on a high level^15^, it seems reasonable why the majority of the general population do not know the importance of BLS. It is also likely that the majority of the general adult population do not have any idea of what BLS is. Therefore, BLS training should start in public education to ensure broader population access.

The strength of this study is that it is the first study conducted on BLS knowledge and practice among medical professionals in Afghanistan. The findings from this study should alert policymakers to pay attention to the lack of BLS knowledge among medical professionals and encourage campaigns and training programs to improve BLS knowledge. This study also has several limitations. First, the present study was only conducted in the capital, Kabul. It is likely that there are larger deficits in knowledge across the country and specifically in rural areas. A further study from different parts of the country can add more insights on this topic. Second, this study was only a survey and did not include a practical test of BLS competency. Future studies should investigate the best ways to train medical providers in Afghanistan. Additionally, a future study should research the level of knowledge and practice of BLS in local communities.

## Conclusion

BLS knowledge and practice in Afghanistan are very low as evidenced in the findings of this study. Regular BLS courses and re-certification are required to improve the knowledge and practice of the medical professionals to prepare them to respond to medical emergencies effectively in order to improve care for the people of Afghanistan.

## Ethical approval

The study was conducted in accordance with the Declaration of Helsinki. All participants agreed to participate before filling the questionnaire. This study was approved by the institutional ethics committee of Ariana Medical Complex (AMC-RC-31-20211208).

## Consent

NA.

## Source of funding

None.

## Author contributions

All authors made a significant contribution to the work reported, whether that is in the conception, study design, execution, acquisition of data, analysis, and interpretation, or in all these areas; took part in drafting, revising, or critically reviewing the article; have agreed on the journal to which the article has been submitted; and agree to be accountable for all aspects of the work. All authors attest they meet the ICMJE criteria for authorship.

## Conflicts of interest disclosure

None.

## Research registration unique identifying number (UIN)

1. Name of the registry: NA.
2. Unique identifying number or registration ID: NA.
3. Hyperlink to your specific registration (must be publicly accessible and will be checked): NA.

## Guarantor

Arash Nemat.

## Provenance and peer review

Not commissioned, externally peer reviewed.
